# Effect of Ultrasonic Parameters on Electrochemical Chloride Removal and Rebar Repassivation of Reinforced Concrete

**DOI:** 10.3390/ma12172774

**Published:** 2019-08-29

**Authors:** Qingyang Liu, Zijian Song, Huanchun Cai, Aiping Zhou, Wanyi Wang, Linhua Jiang, Yongqi Liu, Yingjie Zhang, Na Xu

**Affiliations:** 1 College of Mechanics and Materials, Hohai University, Xikang Road 1#, Nanjing 210098, China; 2 College of Agricultural Engineering, Hohai University, Xikang Road 1#, Nanjing 210098, China; 3 College of Rail Transit, Nanjing Vocational Institute of Transport Technology, Longmian Ave 629#, Nanjing 211188, China

**Keywords:** ultrasonic, ECR, corrosion of steel bars, energy conservation, reduce waste

## Abstract

Electrochemical chloride removal (ECR) from reinforced concrete can be considered as an environment-friendly technique since it can reduce the environmental issues arising from demolition and reconstruction. In this study, we used ultrasonic waves to promote the ECR efficiency without increasing the current density so as to shorten the overall power-on time, lowering the power consumption and electricity-induced material damage. Rebar-embedded cement mortar specimens were prepared and a set of ultrasonic-assisted ECR test devices was designed. For obtaining the optimal parameters, different ultrasonic frequencies and powers were adopted to conduct the ECR test. After that, the discharged and residual chloride ion amounts were detected to characterize the ECR efficiency. The corrosion behavior of rebar was characterized by electrochemical method. It was found that ultrasonic waves can not only promote the discharge of chloride ions, but also promote the passivation process of steel bar. For this investigation, the ultrasonic waves with a frequency of 40 Hz and a power of 60 W had the best auxiliary effect and could reduce the work time by 64%. It is considered that the ultrasound-assisted method has potential to promote the application possibilities of the ECR technique.

## 1. Introduction

Reinforced concrete structures are the most common and widely-used construction forms in the modern world. However, reinforced concrete is vulnerable to chloride-induced corrosion in coastal, salt lakes and deicing salts environments [1,2,3]. With chloride ions migrating into the interior of reinforced concrete, the passive film on the rebar surface will be rapidly destroyed, finally resulting in severe rebar corrosion [4]. Chloride-induced corrosion is one of the main reasons for the untimely failure of reinforced concrete structures. In the past, many structures had to be demolished and reconstructed due to this problem, resulting in huge economic loss and resource consumption [5,6,7]. Moreover, the demolition and reconstruction also produced a large number of urban solid wastes, further resulting in many environmental issues. Thus, it is significant to study the efficient rehabilitation methods of reinforced concrete structures serving in chloride-dense environments or polluted by chloride salts.

The traditional rehabilitation method is to knock out the polluted concrete and replace it with new mortar or concrete. Although this method is simple and relatively fast, due to the difference of chloride ion content and alkalinity between the old and new materials, it is possible to form new corrosive galvanic cells at the alternation of the old and the new, resulting in re-corrosion of steel bars [8,9]. In addition, this method cannot be carried out at the same time as the normal use of the structure. In order to better deal with this problem, electrochemical chloride removal (ECR) technology was proposed by the Federal Highway Administration of the United States, and soon found some engineering applications in North America, Europe and Japan [10]. This method is not only a non-destructive remediation method, but also can remove the invaded chloride ions from the source, so it has attracted extensive attention of researchers. However, the current ECR efficiency was still insufficient, and the ECR process and repassivation process needed a long duration (usually 40–60 d) since much time was wasted in waiting for the transformation of bound chlorides to free ones. Subsequently, much of the electrical power was consumed during the long electrification process, and more negative effects, e.g., hydrogen embrittlement, emerged [11,12]. Therefore, it is urgent to find an appropriate way to promote ECR efficiency. 

Recently, Elsener et al. [13] and Velu et al. [14] proposed an intermittent power-on ECR method which increased the chloride removal efficiency significantly. This method utilized the contrived power-off time to increase the releasing of free chloride ions from bound ones. From our viewpoint, however, it is better to take an active approach to accelerate chloride release rather than passively waiting. Getting inspiration from this, we decided to use ultrasonic waves to assist ECR knowing they could accelerate most of the chemical and physical processes [15]. In fact, ultrasonic waves have been widely used in the nondestructive tests in reinforced concrete structures, providing a circumstantial evidence of the feasibility of this technique. However, relevant studies to ECR have rarely been reported. Therefore, this paper attempts to use the ultrasonic waves to improve the ECR technique so as to promote the application possibilities of this technique.

## 2. Experimental Procedures 

### 2.1. Materials and Specimen Preparation

**Figure 1:**
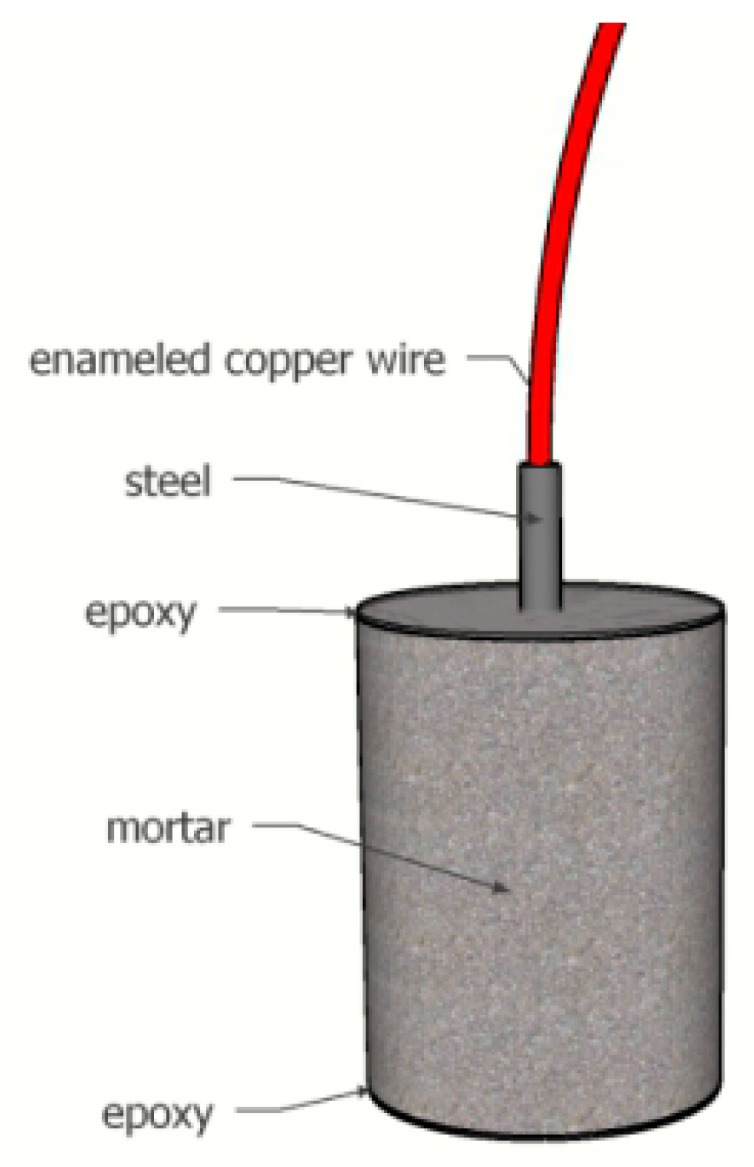
Rebar-embedded cement mortar specimen.

Cement was P·II 42.5 Portland cement produced by China Cement Plant. Its main chemical composition is shown in Table 1. The aggregate was natural river sand with a fineness modulus of 2.8 from Nanjing, China. Cylindrical mortar specimens (75 mm in diameter and 100 mm in length) were prepared with a water-to-cement ratio of 0.5 and a cement-to-sand ratio of 1:2.5. Sodium chloride was mixed in the specimens in a content of 3% by mass of cement. The flowability of the cement mortar was 186 mm, which was determined following ASTM C1437-15. A HPB235 steel bar, 8 mm in diameter and 130 mm in length, was embedded along the central axis of the cylindrical specimen. The two ends of the steel bar were sealed with epoxy resin and then protected by heat shrinkable tubes. One of the two ends was welded with an enameled copper wire inside the shrinkable tube. The middle part with a 30 mm length was set as the exposure surface. Before being embedded, the steel bar underwent a surface pretreatment (polished with 180–2000 grade silicon carbide sandpaper and washed with acetone and absolute ethanol). Then the steel bars were dried naturally and embedded into mortars via a removable stick holder. One day later, the rebar-embedded specimens were demolded and then sealed with epoxy resin on the upper and lower surfaces, which is schematically shown in Figure 1. Then they were immediately placed in the standard curing room (20 ± 2 °C, humidity >95%) for 28 days’ curing.

### 2.2. Ultrasonic-Assisted Electrochemical Treatment

**Figure 2:**
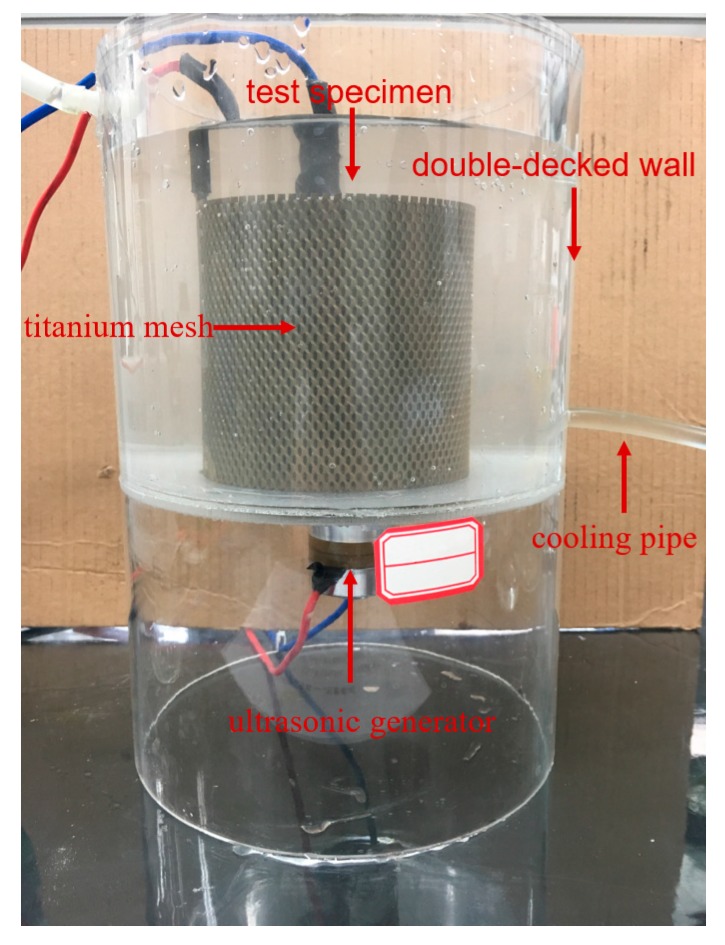
Ultrasound-assisted electrochemical chloride removal (ECR) device.

An electrolytic cell with a diameter of 150 mm and a height of 150 mm was designed and manufactured with polymethyl methacrylate. In the cell, a ring-shaped titanium mesh, 80 mm in diameter and 100 mm in height, was employed as the anode to connect the power supply. The embedded steel bar was used as the cathode. The ultrasonic generator was set at the bottom of the electrolytic cell. Owing to the small size of the electrolytic cell, the temperature of the solution will be uncontrollable under the thermal effect of long-term ultrasound, which is inconsistent with the actual application scenario. Thus, the cell wall was designed as double-decked, the cooling liquid could pass the interlayer to control the temperature of the system, and the ultrasonic generator worked ten seconds an hour. An actual ECR device is shown in Figure 2, and we made six ECR devices with different ultrasonic generators for this study. After assembling the test devices, the ECR experiment could be started. In this study, the average current density was set to 2 A/m^2^ through the surface area of the steel bar. At the same time, the current was monitored every day and stabilized by adjusting the resistor. The saturated Ca(OH)_2_ solution was used as anolyte, which was refreshed every 3 days. The cooling liquid was tap water.

### 2.3. Measurements of Discharged and Residual Chloride Quantities

Before each solution refresh, the chloride ion quantity of the anolyte solution was determined via potentiometric titration and then recorded. The total discharged chloride ion quantity can be calculated by summing the recorded chloride ion quantities. 

**Figure 3:**
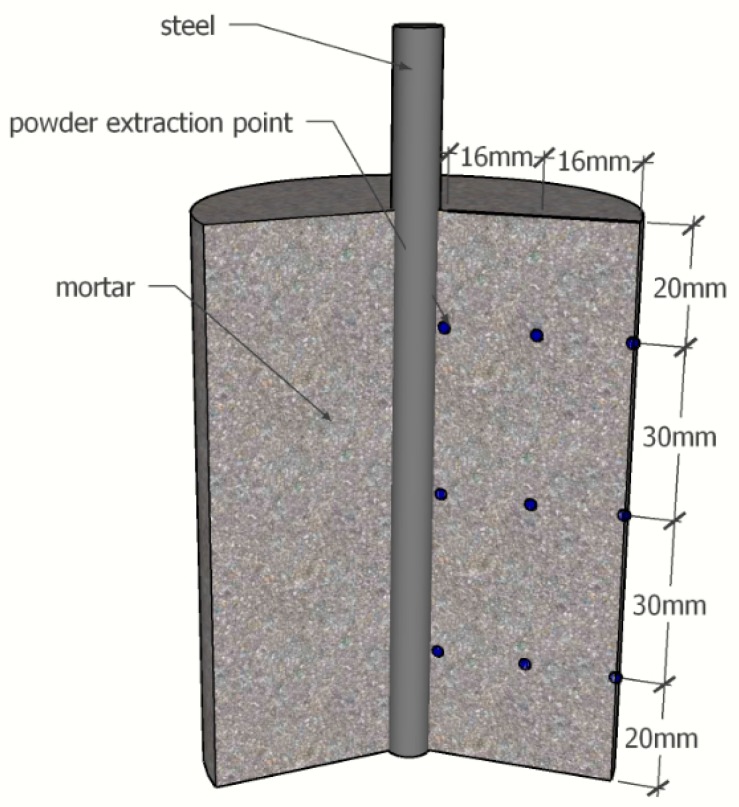
The location of powder extraction.

The specimens with and without ECR were drilled to collect powder to measure the chloride ion distribution. The location of powder sampling was shown in Figure 3. The obtained powder was weighed at 2.000 g and soaked in 40 mL deionized water or 40 mL dilute nitric acid solution (the volume ratio of concentrated nitric acid to deionized water was 15:85) for measuring free and total chloride ion content, respectively. After soaking, 10 mL filtrate solution was taken out. Before titration, the filtrate solution for free chloride ions needed to be acidified to exclude the influence of hydroxide. Then the chloride ion contents were tested via an automatic potentiometric titrator using silver nitrate (AgNO_3_) as titrant. The free chloride ion content was calculated according to Equation (1):

(1) $$C_{f}=\frac{0.03535\times C_{AgNO_{3}}\times V_{3}}{G\times \frac{V_{2}}{V_{1}}}\times 100$$

where, *C_AgNO__3_* is the concentration of silver nitrate solution, *G* is the mass of mortar specimen, *V*_1_ is the volume of deionized water when soaking mortar, *V*_2_ is the volume of filter solution when titrating, and *V*_3_ is the volume of silver nitrate consumed in titration. In the case of calculating the total chloride ion quantity, Equation (1) is still applicable but the *V*_1_ becomes the volume of the dilute nitric acid used for soaking.

### 2.4. Electrochemical Test 

For characterizing the repassivation behavior of the rebar, open circuit potential (OCP) and electrochemical impedance spectroscopy (EIS) were conducted via a PARSTAT 2273 multifunctional workstation. The three-electrode system was used for the electrochemical test, including the steel bar as working electrode, saturated calomel electrode as reference electrode, and platinum electrode as auxiliary electrode [16,17].

A five-minute OCP test was implemented to measure the corrosion potential (*E_corr_*). The EIS scanning was carried out by applying a sinusoidal potential perturbation of 10 mV with a frequency scan from 100 kHz to 0.01Hz. Using ZSimpWin software, the measured spectra were fitted and sorted out, and the values of the polarization resistances (*R_p_*) of steel bar were obtained. Then the corrosion current (*I_corr_*) of steel bar can be calculated by Equation (2) (i.e., the Stern–Geary Equation) [18]. Since *I_corr_* is related to the exposed surface area of the steel electrode, the corrosion current density (*i_corr_*) is usually used to normalize the corrosion rate, which can be calculated by Equation (3).

(2) $$I_{corr}=\frac{B}{R_{p}},$$

(3) $$i_{corr}=\frac{I_{corr}}{S}$$

Among them, *S* is the side surface area of steel (25.13 cm^2^ in this study), *B* is the Stern–Geary constant (the values are different according to the different states of steel bars). According to the literature, *B* can be approximately equal to 26 mV when the steel bar is activated and 52 mV when the steel bar is passivated [19,20].

## 3. Results and Discussion

### 3.1. Effect of Ultrasound Parameters on Chloride Ion Discharge

**Figure 4:**
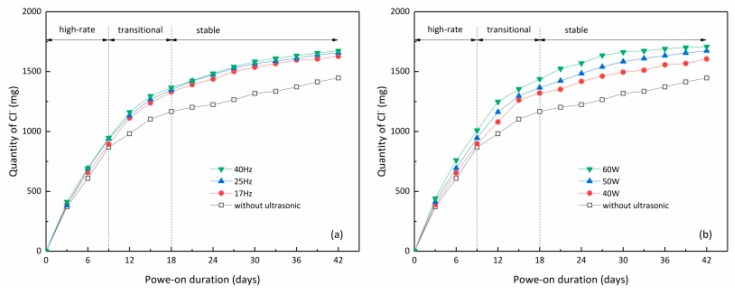
Variation of chloride ion discharge with time in ECR process under different ultrasonic parameters: (**a**) different ultrasound frequencies, (**b**) different ultrasound powers.

The discharged chloride ion quantity is an important value to intuitively present the ECR efficiency. Figure 4 shows the variation of the discharged chloride ion quantity during ECR tests with and without ultrasonic assistance. 

As can be seen from Figure 4, the discharged chloride ion quantity generally increased rapidly at the initial stage of ECR tests. However, with the prolongation of ECR, the discharge rate of chloride ions (seen from the slope of the variation curve) gradually slowed down and finally reached a relatively small level. To further elaborate the detailed mechanisms, we generally divided the variation curves into three stages, including the high-rate stage (i.e., the initial stage, about the first nine days), the transitional stage (about the second nine days) and the stable stage (after about the 18th day). The emergence of the transitional stage can be attributed to the lack of free chloride ions at this stage. It is known that the electric field can only make free chloride ions discharged. In the initial stage, there were sufficient free chloride ions inside the specimens to ensure the high discharge rate. With the extension of ECR duration, the free chloride ions inside the specimen have been greatly discharged. From the kinetics point of view, it would take certain time to reach the new equilibrium between bound chloride ions and free ones. The rate of converting bound chloride ions to free ones cannot catch up with the rate of chloride ion discharge [21]. Thus, the free chloride ions at this stage would be insufficient to support a high ECR efficiency, manifesting as a gradual slow-down of the discharge rate. Hence, the transitional stage emerged. 

From Figure 4 it can be found that for any duration the discharged chloride ion quantity of ultrasonic-assisted ECR treatment was greater than that of the non-ultrasonic one, indicating that ultrasound had a positive effect on the electrochemical chloride removal. This is possibly due to the fact that ultrasound can promote the release of bound chloride ions to free ones, knowing that ultrasound has a favorable impact on the rate of most of the physicochemical processes by producing a series of mechanical, thermal, electromagnetic and chemical effects [16,22]. Later studies on chloride ion binding rate also validated this conjecture. It should be noted that, in the initial stage, the chloride ion discharge rates for the ultrasonic-assisted ECR treatment were only a bit higher than those for the non-ultrasonic one, but this gap became greater in the transitional stage. This was also consistent with the previously mentioned mechanism since it can be easily deduced that the chloride-release effect of ultrasonic assistance was more pronounced in the case of less free chloride ions.

Figure 4a presents the discharged chloride ion quantities in ECR processes under different ultrasonic frequencies. It can be seen from the figure that the ultrasonic frequency had a modest effect on the discharge efficiency within the region from 17 Hz to 40 Hz. When the power was fixed at 50 W, the discharged chloride ion quantities for the ultrasonic frequencies of 17 Hz, 25 Hz and 40 Hz after 42 days of power-on were 1627 mg, 1658 mg and 1673 mg, respectively. The discharged quantity had a weak positive correlation with the ultrasonic frequency. The reason is assumed as follows. When an ultrasound acts on medium, it can produce an acoustic relaxation phenomenon, which makes the energy freely transfer between molecules, macroscopically manifesting as the change of chemical properties and structure of substances [23]. When an ultrasonic wave is penetrating a concrete medium, it can produce strong mechanical vibration impact and cavitation on the interface between liquid and solid [24]. The high-frequency ultrasonic wave has short wavelength, making it more apt to be blocked by concrete. Hence, more power is captured, which can better promote the transformation of bound chloride ion to free chloride ion in concrete. On the other hand, due to the cavitation of high-frequency ultrasound, the number of bubbles on the surface of the titanium mesh is greatly reduced, which ensures that the current can pass through the electrode smoothly, improves the moving speed of charged ions, reduces the polarization of the electrode reaction concentration, and improves the current efficiency.

Figure 4b shows the discharged chloride ion quantities in ECR processes under different ultrasonic powers. It can be clearly seen that the discharge efficiency remarkably increased with the ultrasonic power. When the frequency of ultrasound was 40 Hz, the 42-day discharged chloride ion quantities of ECRs with ultrasonic powers of 40 W, 50 W and 60 W were 1627 mg, 1658 mg and 1673 mg, respectively. But this does not mean that we could unlimitedly use the high-power ultrasound-assisted ECR since the exorbitant ultrasonic power is more apt to damage the concrete microstructure, thereby degrading the durability of the whole structure. Further studies on this issue should be taken. In terms of chloride removal efficiency, the 60 W/40 Hz ultrasonic wave has the best auxiliary effect. Its discharged chloride quantity on day 15 was similar with the non-ultrasonic ECR on day 42. This means that it can shorten the power-on time by 64%, lowering the electricity-induced material damage (e.g., hydrogen embrittlement) and the overall power consumption.

### 3.2. Effect of Ultrasonic Parameters on Residual Chloride Ion Content After ECR

**Figure 5:**
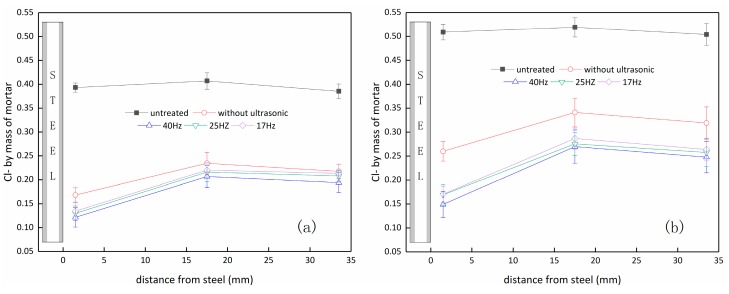

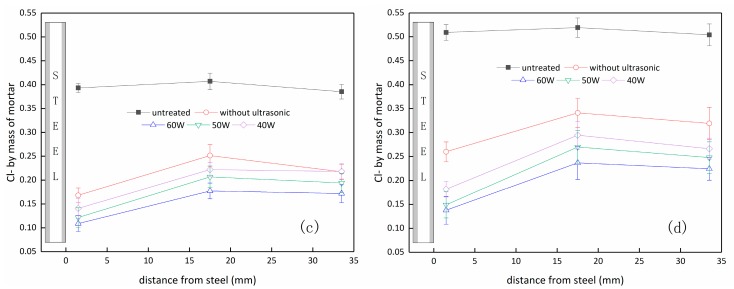
The distribution of chloride ion residues. (**a**) Free chloride ions at different ultrasound frequencies; (**b**) total chloride ions at different ultrasound frequencies; (**c**) free chloride ions at different ultrasound powers; (**d**) total chloride ion at different ultrasound powers.

Figure 5 showed the distributions of chloride ion residues in cement mortar specimens after ultrasonic-assisted ECR treatment, non-ultrasonic ECR treatment and without ECR treatment. It can be seen that both the free chloride ions and the total chloride ions were uniformly distributed in the specimens without ECR treatment, and their contents were about 0.395% and 0.511% (by mass of mortar), respectively. The contents of chloride ions, either free or bound, in cement mortars after ultrasonic-assisted ECR treatments were lower than those after non-ultrasonic ECR treatments or without ECR treatment, which further illustrated that the ultrasonic-assisted electrochemical method could promote the discharge of chloride ions from the specimens. It should be noted that this promotion effect was more reflected in the reduction of residual total chloride ions. For example, the residual total chloride ions for 40 Hz/60 W ultrasonic–assisted ECR treatment were 35% lower than that for non-ultrasonic ECR treatment, while the residual free chloride ions were only 28% lower comparing with the non-ultrasonic ECR treatment. This further illustrated that the promotion of ultrasonic waves on ECR efficiency was mainly from the increase of the transformation of bound chloride ions to free ones, which might be another merit of ultrasonic-assisted ECR technique since there is no need to over worry about the potential risk caused by the re-conversion of bound chloride to free ones after the process.

Generally, it can be found that the distribution of residual chloride ion content varies with ultrasonic parameters. Figure 5a,b shows the effect of different frequencies. It is found that both the free and the total chloride ion content in the specimens are the lowest at the frequency of 40 Hz, which is consistent with the rule for the discharged chloride ion quantity. As previously mentioned, the ultrasonic wave of higher frequency is more apt to be captured. Similarly, Figure 5c,d shows the effect of different powers on the distribution of residual chloride ion content. It can be seen that the residual chloride ion in the specimen significantly decreases with the increase of ultrasonic power, which is also in accordance with the rule for the discharged chloride ion quantity.

It should be noted that the residual chloride ion content in the middle layer was the highest, while the chloride ion content near the steel bar was the lowest. This was mainly because the chloride ion near the cathode region tends to migrate to the area away from the reinforcing steel under the action of electric field force. The residual chloride ion content of the outer layer also became slightly smaller. As we know, the anolyte was frequently refreshed, and the chloride ion content was maintained in very low value. Thus, the chloride ions in the outer layer tended to diffuse into the anolyte under the action of concentration gradient, leading to a content drop in the outer layer.

### 3.3. Effect of Ultrasonic Parameters on Steel Bar Repassivation after ECR

**Figure 6:**
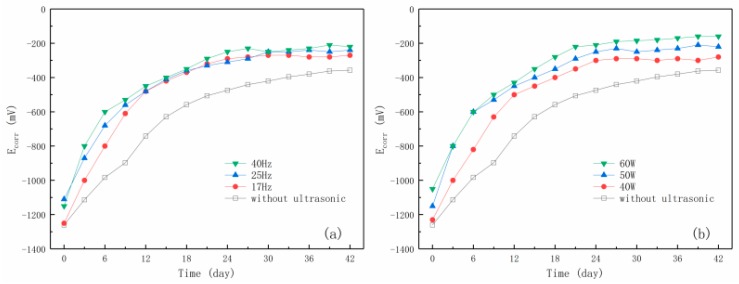
The curves of corrosion potential of steel bar in desalting specimens with time. (**a**) Different ultrasound frequencies; (**b**) different ultrasound powers.

*E_corr_* is one of the important indicators used to characterize the state of a steel bar [24]. Figure 6a,b represents the *E_corr_* evolution curves of the embedding steel bars after ECR treatments assisted by different ultrasonic frequencies and powers, respectively. After ECR treatment, the corrosion potentials of steel bars in the specimens were generally lower than –1000 mV, which was consistent with the findings of many previous researchers [18,25]. As explained in these studies, the reduction of *E_corr_* after ECR is probably due to the polarization of the steel bar connected with the negative pole of the power supply. In the following 42 days after ECR treatment, the *E_corr_* of steel bar first tended to decrease rapidly and then reached a stable and relatively positive level, which can be seen as a result of repassivation. With the increase of depolarization time, oxygen diffuses to the surface of steel bar and produces hydroxide ions, which will make the corrosion potential of steel bar move forward. Comparing with the repassivation process without ultrasonic assistance, it can be obviously found that ultrasonic waves can make the rehabilitation of corrosion potential of reinforcing steel bar faster. This is attributed to the fact that the ultrasonic cavitation could effectively promote the diffusion rate of oxygen to accelerate the repassivation. Besides, as mentioned previously, the ultrasonic waves could further reduce the residual chloride ion content, which provided a better chemical environment for repassivation.

**Figure 7:**
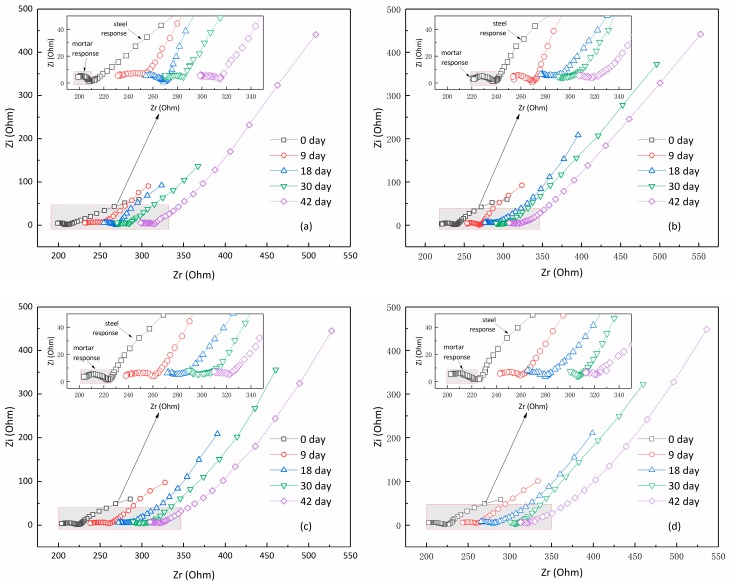
Nyquist spectra at different times. (**a**) Without ultrasound; (**b**) 40 Hz/60 W ultrasound; (**c**) 40 Hz/50 W ultrasound; (**d**) 17 Hz/50 W ultrasound.

In order to further reveal the effect of ultrasonic-assisted ECR treatment on the repassivation behavior, EIS was used to detect the electrochemical characteristics of steel bars. Figure 7 typically shows the Nyquist spectra of the embedding steel bar after different ECR treatments. It can be seen from the figure that the Nyquist plots generally have two arcs. The arc in the high frequency region was referred to as the mortar response, and meanwhile the arc in the low frequency region was termed the steel response. It can be seen that the connection point of the two arcs gradually moved towards the right side as the storage duration increased. According to Song’s three-path microstructure assumption [26], the right shift of connection points indicated the growth of the resistance of continuously connected micro-pores in the mortar. This meant that the microstructure of cement mortars grew denser after ECR treatments, which is probably due to the migration of Ca^2+^ into the specimen during ECR. Ca^2+^ will gradually deposit in the pores of cement mortars, resulting in the densification of mortars [25]. The Nyquist plots at low frequencies reflect the capacitance and resistance of the steel bar. The arc diameters at high frequencies generally increased with the duration, which indicated that the passivation film gradually formed on the surface of steel bar.

**Figure 8:**
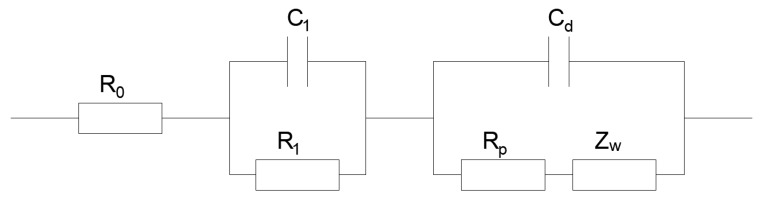
Typical equivalent circuit of reinforced concrete structure.

For quantitatively analyzing the impedance spectra, an electrical equivalent circuit mode (ECM) was adopted to fit the response signal as shown in Figure 8. This equivalent circuit can be described by CDC (Circuit Description Code) as *R*_o_(*C*_1_*R*_1_) (*C_d_*(*R_p_Z_w_*)). In the circuit, the left part, i.e., the terms of *R*_o_(*C*_1_*R*_1_) represents the response of cement mortar. The left part can be considered as a simplified equivalent model for Song’s three-path ECM [26].

(4) $$R_{0}=R_{CP}R_{CCP}/(R_{CP}+R_{CCP}),$$

(5) $$R_{1}=R^{2}{}_{CCP}/(R_{CP}+R_{CCP}),$$

(6) $$C_{1}=1/2{\left(1+R_{CP}/R_{CCP}\right)}^{2}C_{DP}+{\left[C^{2}{}_{DP}-4C_{DP}C_{mat}/{\left(1+R_{CP}/R_{CCP}\right)}^{2}\right]}^{1/2},$$

where, *R_CCP_* is the resistance of the continuously connected micro-pores in the concrete; *R_CP_* is the resistance of the discontinuously connected micro-pores; *C_mat_* is the capacitance across the concrete matrix; *C_DP_* is the capacitance of the cement paste layers blocking the discontinuously connected micro-pores in the concrete. According to this, *R*_0_ + *R*_1_ is equivalent to R_CCP_, whose value is approximately equal to the abscissa value of the connection point of high-frequency arcs and low-frequency arc in the Nyquist plot, as previously mentioned. The right part of the used ECM, i.e., the terms of (*C_d_*(*R_p_Z_w_*), represents the response of steel bar, where, *C_d_* is the double layer capacitance of concrete/steel interface; *R_p_* is the reactive charge transfer resistance of steel surface electrode; *Z_w_* is the Warburg impedance related to diffusion process. 

**Figure 9:**
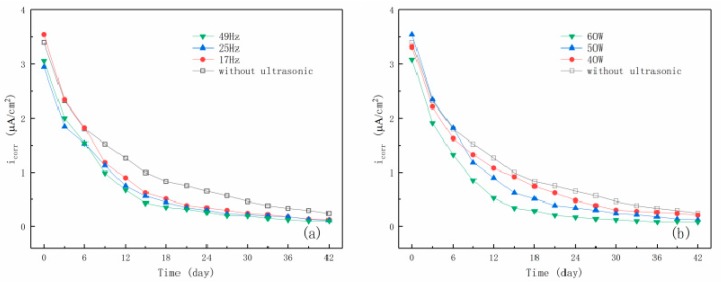
The curves of current density of steel bar in desalting specimens with time. (**a**) Different ultrasound frequencies; (**b**) different ultrasound powers.

Using the ZSimpWin software, the measured spectra were fitted and sorted out, and the values of the *i_corr_* of steel bar were obtained. Figure 9a,b shows the curves of *i_corr_* after ECR assisted by ultrasonic waves with different frequencies and powers. It can be seen from the figure that the *i_corr_* of steel bar had exceeded 3 μA/cm^2^ within a short time after ECR treatment. This current increase is mainly due to the polarization voltage of the steel bar connected with the negative pole of the power supply. In the following 42 days, the *i_corr_* of steel bar first decreased rapidly and then reached a relatively stable and small level, which declared that the corrosion has been effectively controlled due to the repassivation. As previously mentioned, as depolarization time developed, oxygen diffused to the surface of steel bar to produce hydroxide ion, and meanwhile chloride ions were pushed away from the steel surface, which provided a good environment for repassivation. Comparing with the repassivation process without ultrasonic assistance, it can be obviously found that ultrasonic waves can make the decline of the corrosion current density faster. As mentioned previously, this is attributed to the fact that the ultrasonic cavitation could effectively promote the diffusion rate of oxygen and discharge rate of chloride ions. Besides, as reported [27], the water molecules will be decomposed into hydrogen radicals and hydroxyl radicals, which form hydrogen and hydrogen peroxide, which can accelerate the recovery of passive film on steel surface.

## 4. Conclusions

In this study, we used ultrasonic waves to promote the ECR efficiency without increasing the current density. On the basis of the results and discussion above, the following conclusions can be drawn:

(1). Ultrasonic waves could effectively promote the ECR efficiency. When frequency is located in 17–49 Hz and power is in 40–60 W, the promotion effect has a weak positive correlation with the ultrasonic frequency and a relatively strong positive correlation with the ultrasonic power.
(2). The promotion of ultrasonic waves on ECR efficiency was more reflected in the reduction of residual bound chloride ions, which might be a merit of ultrasonic-assisted ECR technique since it would reduce the potential risk from the prospective re-conversion of bound chloride to free ones after ECR treatment.
(3). Electrochemical tests demonstrated that ultrasonic-assisted ECR treatment could accelerate the repassivation process of the embedding steel bar.

It is considered that the ultrasound-assisted method has the potential to promote the application possibilities of the ECR technique, and the applications of this technique in practical engineering may be one of the urgent topics for follow-up research.

## Figures and Tables

**Table 1:** Main chemical composition of P·II 42.5 cement.

CaO	SiO_2_	Al_2_O_3_	Fe_2_O_3_	SO_3_	MgO	LOI	K2O	Na_2_O	TiO_2_	MnO_2_	P_2_O
64.65	21.70	5.08	4.32	1.08	0.92	0.85	0.54	0.22	0.14	0.10	0.05
